# Risk assessment of radionuclides in different environmental matrices in the State of Qatar, Arabian Gulf

**DOI:** 10.1007/s10653-026-03469-1

**Published:** 2026-09-09

**Authors:** S. Veerasingam, V. Vinothini, M. Ranjani, Rania Edrees Adam Mohammad, G. Suresh, S. Rajendran, Kishor Kumar Sadasivuni, Saud Ghani, Fatima Al-Khayat

**Affiliations:** 1 Environmental Science Center, Qatar University, P.O Box 2713, Doha, Qatar; 2 Department of Humanities and Sciences/Physics Division, Aarupadai Veedu Institute of Technology, Vinayaka Mission’s Research Foundation (DU), Payanoor, Chennai, Tamil Nadu 603104 India; 3 Center for Advanced Materials, Qatar University, PO Box 2713, Doha, Qatar; 4 Mechanical and Industrial Engineering, Qatar University, 2713, Doha, Qatar; 5 Department of Biological and Environmental Sciences, College of Arts and Sciences, Qatar University, Doha, Qatar

**Keywords:** Environmental radioactivity, Natural radionuclides, Artificial radionuclides, Risks, Qatar

## Abstract

Environmental radioactivity in Qatar arises from a combination of naturally occurring radionuclides (NORM) and technically enhanced NORM (TENORM) associated with industrial activities. This study presents a comprehensive systematic review with quantitative synthesis of radionuclide concentrations across multiple environmental matrices, including soil, sediment, groundwater, seawater, building materials, and marine biota, extracted from research papers published between 1980 and September 2025. Data synthesis reveals those natural radionuclides such as ^226^Ra, ^238^U, ^232^Th, and ^40^K exhibit moderate spatial variability, influenced primarily by lithology, soil texture, and arid climatic conditions. In contrast, artificial radionuclides (^137^Cs, ^90^Sr) are detected at trace levels, consistent with global fallout. Radiological risk assessment using indices such as radium equivalent activity, absorbed dose rate, annual effective dose, gonadal dose equivalent, and hazard indices indicates minimal risk for most soils, sediments, and conventional building materials. However, elevated activity concentrations and associated risk metrices are observed in industrial by-products, oil-field residues, and waste materials highlighting localized hazards. Key sources include geological formations, dust storms, hydrocarbon extraction, construction materials, and minor transboundary inputs. Despite the overall low exposure, continuous monitoring, strict management of industrial residues, and regional cooperation are recommended to mitigate potential health and environmental risks. This systematic review with quantitative synthesis provides a robust baseline for future environmental radioactivity research and policy development in arid regions.

**Supplementary Information:**

The online version contains supplementary material available at https://doi.org/10.1007/s10653-026-03469-1.

## Introduction

Naturally occurring and anthropogenic radionuclides are ubiquitous in the Earth’s crust, contributing to the primary sources of background radiation in different matrices, including soil, water, air, and biota. The natural radiological background mainly arises from the decay series of primordial radionuclides such as uranium 238 (^238^U), thorium 232 (^232^Th) and potassium 40 (^40^K) along with their progeny, including radium isotopes (^226^Ra and ^228^Ra). Artificial radionuclides are radioactive isotopes produced through human activities rather than by natural geological processes. Unlike naturally occurring radionuclides, artificial radionuclides are generated primarily through nuclear fission, neutron activation, or particle bombardment in nuclear reactors, accelerators, and medical facilities. Since the mid-twentieth century, these radionuclides have become important environmental contaminants due to atmospheric nuclear weapons testing, nuclear power generation, accidental releases from nuclear facilities, medical isotope production, and various industrial applications. Atmospheric nuclear weapons testing conducted between 1940 s and 1980 introduced substantial quantities of fission products into the global environments. Radionuclides, such as cesium 137 (^137^Cs), strontium 90 (^90^Sr), and Plutonium 239 (^239^Pu) were injected into the stratosphere and subsequently dispersed worldwide through atmospheric circulation before being deposited onto terrestrial and marine ecosystems through precipitation and dry fallout. Although atmospheric testing was largely discontinued following the Partial Nuclear Test Ban Treaty of 1963, these radionuclides continue to persist in the environment because of their relatively long half-lives, particularly ^137^Cs and ^90^Sr. (Abbasi et al., 2022; Carvalho et al., 2016). Artificial radionuclides are also intentionally produced in nuclear reactors and cyclotrons for medical applications. Although these radionuclides are generally well regulated, improper handling or disposal of radioactive medical waste may contribute localized environmental contamination. Similarly, several artificial radionuclides are employed in industrial applications, including non-destructive testing, industrial radiography, thickness, and density gauges, level measurement systems, oil and gas well logging, and scientific research. Systematic monitoring of these radionuclides is essential to understand their environmental behavior, geochemical cycling, and exposure pathways. Geochemical cycling describes the movement and transformation of radionuclides among soil, water, sediment, atmosphere, and biota, whereas exposure pathways refer to the mechanisms by which radionuclides reach humans or ecosystems through inhalation, ingestion, or external irradiation (Alshahri, 2019; Aziman et al., 2025).

The Arabian Gulf region represents a unique radiological environment due to its arid climate, low soil organic content, high salinity, and heavy reliance on desalination for water supply (Mihalik et al., 2024). The distribution and transport of radionuclides across terrestrial and marine environments are further influenced by frequent dust storms, emissions and discharges associated with intensive oil and gas extraction and other industrial activities (Abbasi et al., 2021). In such arid ecosystems, radionuclides are less prone to leaching and often tend to accumulate in surface soils, sabkha sediments, and nearshore marine environments. Moreover, rapid industrialization, urbanization, and the expansion of coastal infrastructure in Gulf countries have intensified the need for continuous radiological assessment (Uddin et al., 2020). Despite these pressures, systematic and integrated studies on radionuclide levels across different environmental compartments remain limited in the region.

The state of Qatar, a peninsula projecting into the Arabian Gulf from the northeastern coast of Arabian Peninsula, is characterized by a hot desert climate, sparse vegetation, and carbonate-rich soils derived from Eocene formations such as the Rus and Dammam formations (Rajendran et al., 2021; Veerasingam et al., 2020). The economic growth of Qatar has been driven primarily by its extensive hydrocarbon industry, particularly oil and natural gas extraction, refining, and petrochemical production presents a potential pathway for the introduction of technologically enhanced naturally occurring radioactive materials (TENORM) (Al-Sulaiti et al., 2012). Additional industrial contributors, including cement production, steel manufacturing, construction and large-scale desalination plants are contributing to the complex interplay of natural and anthropogenic radionuclide sources (Al-Kuwari & Hushari, 2019). Furthermore, economic and ecological activities such as offshore oil exploration, shipping, and coastal development within the exclusive economic zone (EEZ) of Qatar further influence the distribution and concentration of radioactive substances (Al-Qaradawi et al., 2015).

This review systematically compiles, synthesizes, and critically evaluates the available data on radionuclide concentrations in the state of Qatar. It provides a comprehensive analysis of different environmental matrices, including terrestrial components (soil and groundwater), marine compartments (seawater, sediments, and biota), and industrially relevant materials (e.g., construction aggregates). The datasets are derived from a systematic review of peer-reviewed literature and relevant national monitoring reports. The present study aims to: (i) compile and synthesize published data on naturally occurring and artificial radionuclides in different environmental matrices in Qatar, (ii) evaluate their spatial distribution and potential radiological risks to humans using internationally accepted radiological indices, (iii) summarize the available information on radionuclide concentrations in marine biota and other biological matrices, (iv) identify the major natural and anthropogenic sources influencing radionuclide distribution, and (v) highlight current knowledge gaps and propose future research priorities and policy recommendations for environmental radiation protection in Qatar. By integrating findings across diverse environmental compartments, this review provides a holistic understanding of Qatar’s radiological landscape and outlines pathways for improved environmental management, risk mitigation, and sustainable policy development.

## Data extraction

**Fig. 1:**
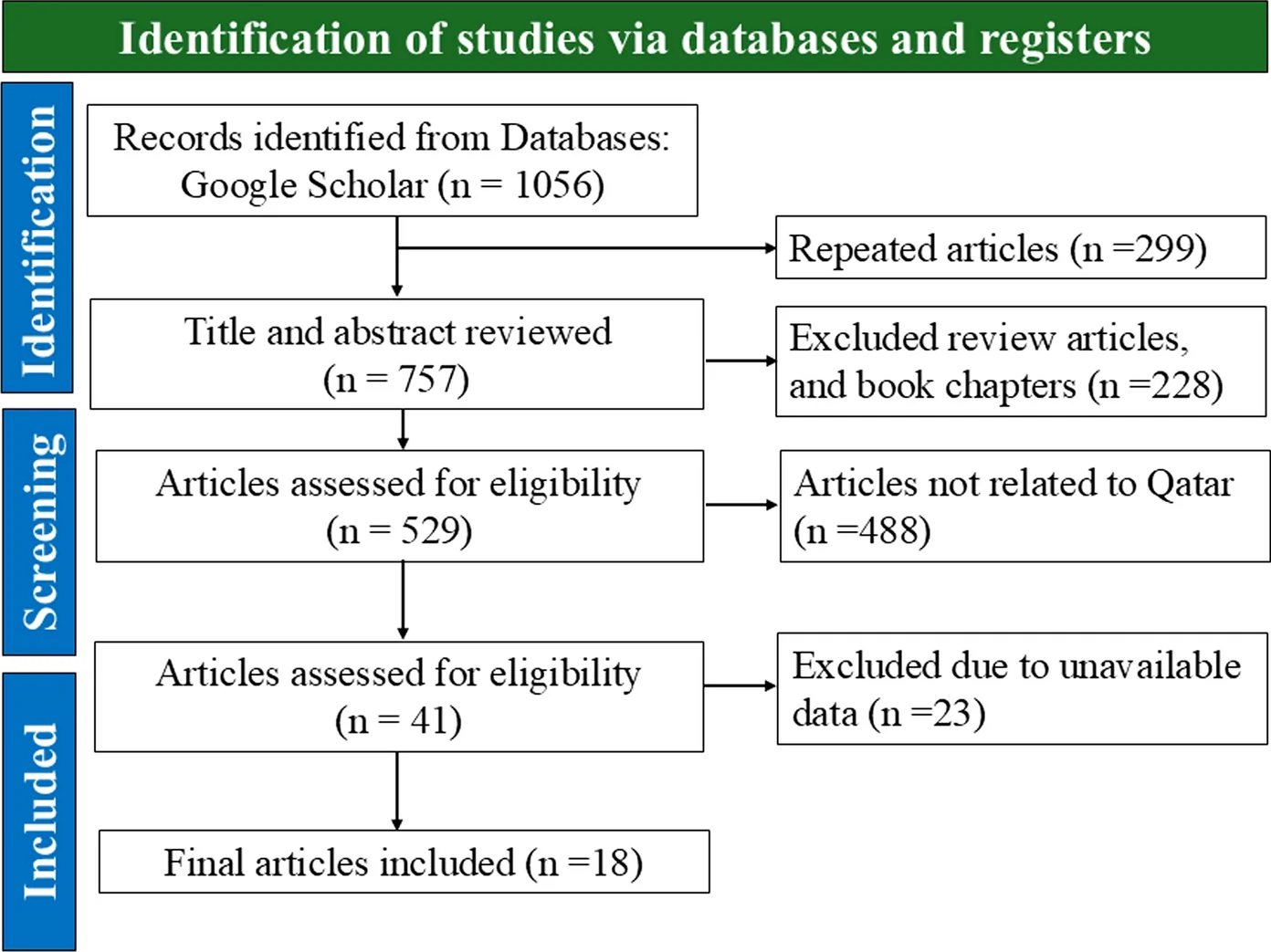
PRISMA method applied in selecting the scientific information included in this work

In this study, a systematic review was conducted following the Preferred Reporting Items for Systematic Reviews and Meta-Analyses (PRISMA) guidelines (Fig. 1). Google Scholar was used as the primary database to identify articles reporting concentrations of natural and artificial radionuclides in different environmental matrices in Qatar from 1980 to September 2025. Web of Science, PubMed, and Scopus were additionally consulted to identify potentially relevant records and cross-check the literature search. The search syntax included the following keywords: “Environmental radioactivity” OR “Radionuclides” OR “Natural radionuclides” OR “Artificial radionuclides” OR “Soil” OR “Sediment” OR “Dust” OR “Building materials” OR “Water” OR “Biota”. Additionally, the reference lists of the selected articles were reviewed to identify further relevant studies. For quantitative data synthesis, studies were included based on the following criteria: (i) availability of the full text in English, (ii) descriptive study design, (iii) reporting of the range and/or average concentration of radionuclides, and (iv) direct relevance to the State of Qatar.

## Environmental matrices and sampling overview

**Fig. 2:**
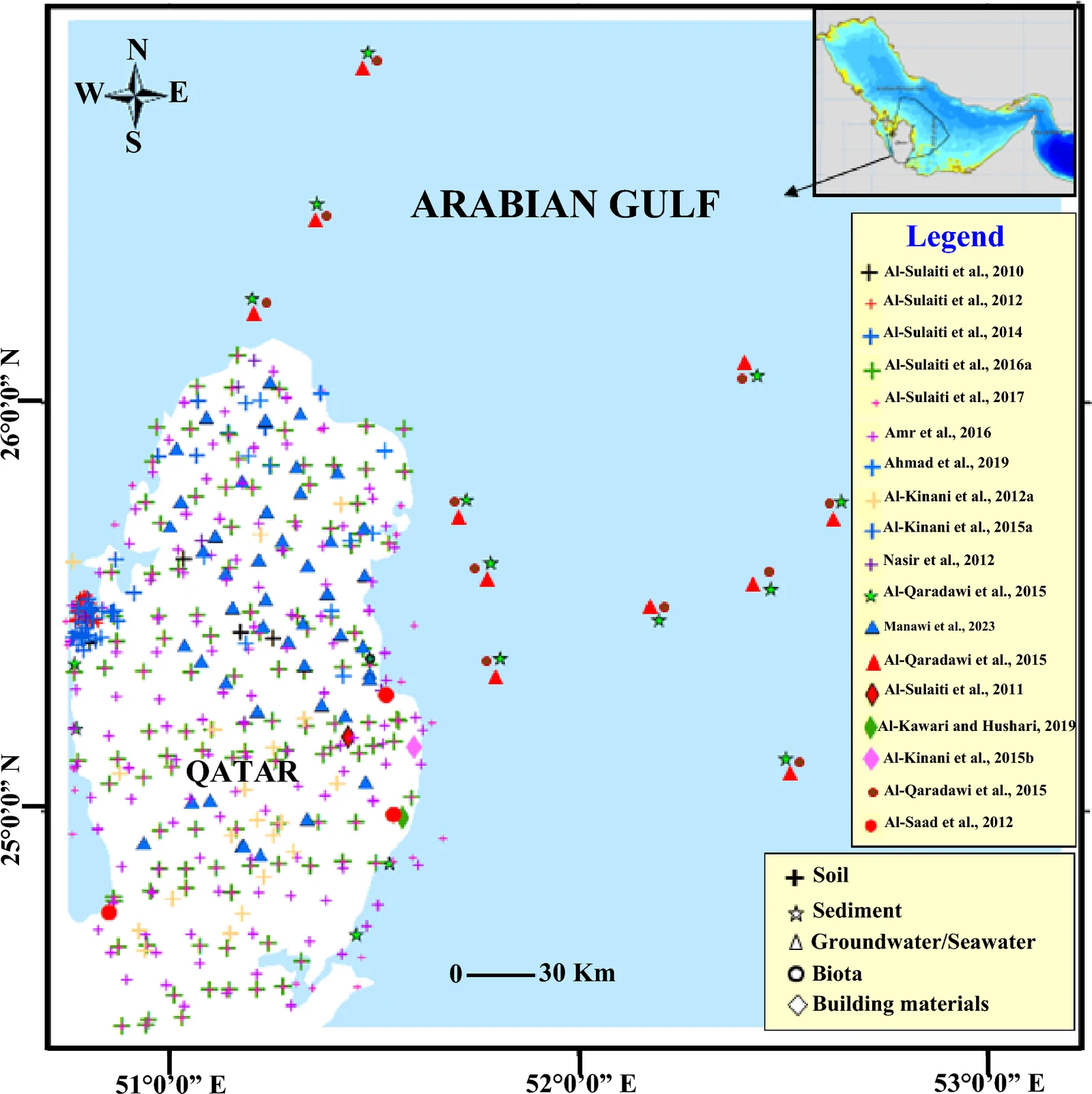
Map showing the radionuclides concentrations in different matrices reported in Qatar

Systematic investigations into environmental radioactivity in Qatar have encompassed a diverse range of matrices, reflecting the diverse geochemical and industrial landscapes of the country. The matrices examined include soil, marine sediments, seawater, groundwater, building materials, and marine biota. These studies have utilized systematic sampling strategies, advanced spectrometric techniques, and robust statistical analyses to characterize the distribution and variability of radionuclides within each environmental compartment. Most studies have been concentrated in the northern and northwestern regions, such as Dukhan (oil field area), Al-Zubara, and Al-Areesh to assess natural radionuclide levels (Al-Sulaiti et al., 2010, 2012, 2014, 2017; Nasir et al., 2012; Al-Kinani et al., 2015a; Ahmad et al., 2019) (Fig. 2). Comparatively fewer studies have investigated other parts of the country (Al-Kinani et al., 2012a, 2012b).

**Fig. 3:**
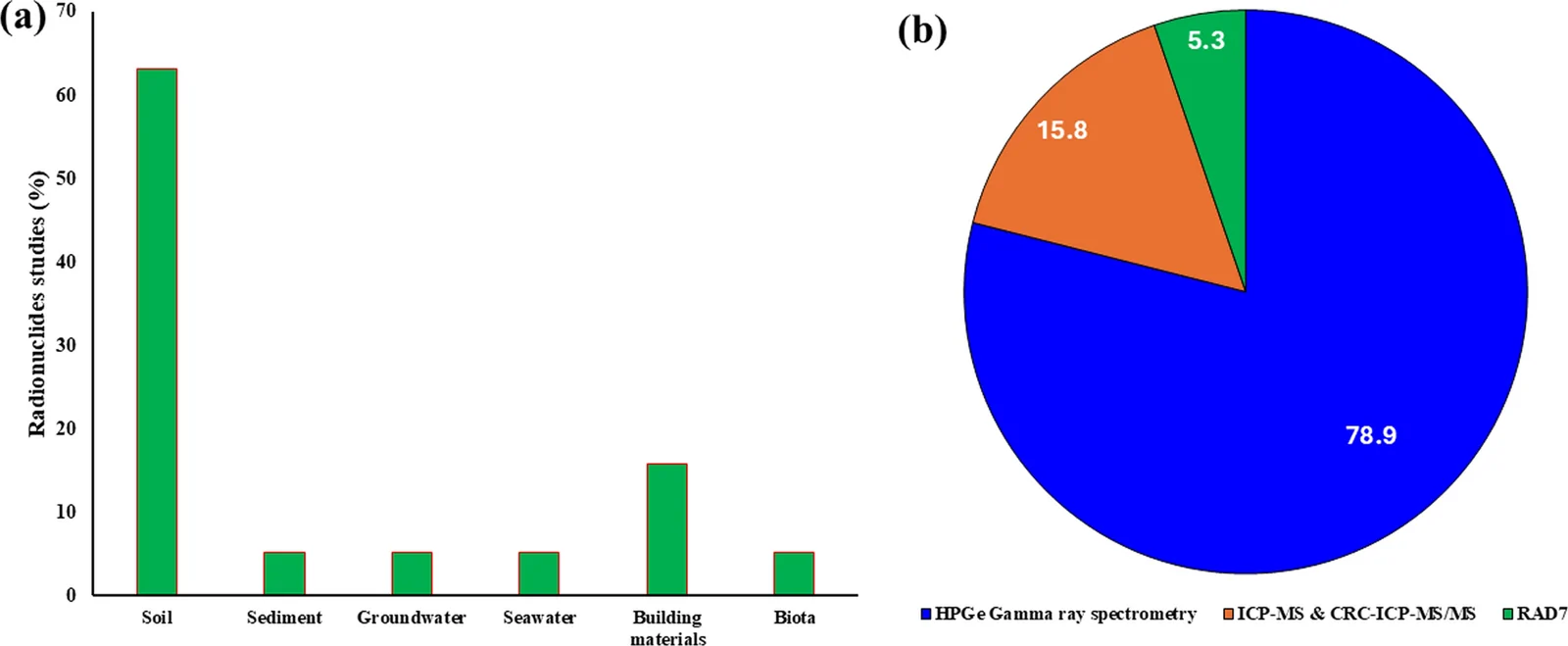
**a** Number of research studies reported radionuclides concentrations in different sampling matrices, and **b** Types of analytical methods applied to assess the level of radionuclides in Qatar

Soil represents the most extensively studied environmental matrix in Qatar, with research covering both surface and subsurface layers across diverse geological and land-use settings (Fig. 3a). The activity concentrations of artificial radionuclides (^137^Cs and ^90^Sr) in soil across Qatar have also been reported in multiple studies (Al-Sulaiti et al., 2016a, 2016b; Amr et al., 2016). In marine environments, Al-Qaradawi et al. (2015) conducted an extensive study on the distribution of both natural and artificial radionuclides in sediments collected from 15 locations within Exclusive Economic Zone (EEZ) Qatar, including nearshore coastal regions. The same study also established the seasonal variations of four natural and one artificial radionuclide in EEZ seawaters. For groundwater, Manawi et al. (2023) investigated ^222^Rn concentrations in samples collected from 48 wells across central, northern, western, and southern Qatar. The widespread use of local industrial by-products in construction has prompted radiological assessments of materials such as cement, concrete, sand, gypsum, steel slag, and asphalt (Al-Houty et al., 1987; Al-Kinani et al., 2015b; Al-Kuwari & Hushari, 2019; Al-Sulaiti et al., 2011). Research on biota has explored the seasonal variations of radionuclides in a wide range of marine organisms, including the pearl oyster (*Pinctada radiata*), bottom-feeding fish (*Lethrinus nebulosus*), shrimp (*Penaeus semisulcatus*), crab (*Portunus pelagicus*), clam (*Dosinia alta*), starfish (*Ophiothrix cf. lineata*), algae (*Sargassum benderi*), sponge (*Haliclona* spp.), sea cow (*Dugong dugon*), and mangrove (*Avecinia marina*) within EEZ of Qatar (Al-Qaradawi et al., 2015). Furthermore, Al-Saad et al. (2012) used sunflower seedlings (*Helianthus annuus *L.) to examine the effects of various chemical agents on the uptake and translocation of depleted uranium.

Radionuclide measurements in these studies were predominantly conducted using high-purity Germanium (HPGe) gamma spectrometry, often complemented by NaI (Tl) detectors for initial screening and dose-rate assessments (Fig. 3b). Other analytical techniques, such as inductively coupled plasma mass spectrometry (ICPMS), Triple Quadrupole Collision/Reaction Cell Inductively Coupled Plasma Mass Spectrometry (CRC-ICP-MS/MS), and RAD7 with RAD H_2_O accessory have also been employed, though to a lesser extent. Sample preparation typically involved drying, homogenization, sieving to a specific grain size, and sealing in Marinelli beakers to achieve secular equilibrium before gamma spectrometric analysis. Subsequent statistical analyses included computation of mean activity concentrations, standard deviations, and various radiological hazard indices to evaluate potential exposure risks.

## Distribution and concentration of radionuclides

The spatial and matrix-specific distribution of radionuclides in Qatar arises from a complex interplay between its unique geological background, intensive industrial activities, and dominant environmental processes, such as aeolian transport and evaporative concentration. Concentrations of naturally occurring radionuclides (^238^U, ^232^Th, ^40^K, ^226^Ra) exhibit moderate variability across soil, sediment, seawater, groundwater, and building materials. In contrast, artificial radionuclides such as ^137^Cs and ^90^Sr are present only at trace or undetectable levels. This section provides a synthesized overview of the observed concentration ranges, delineates key spatial patterns, and offers a critical comparison with international reference values.

### Soil and sediments

Studies of soil samples across the State of Qatar reveal that naturally occurring radionuclides dominate the radioactivity profile with activities generally falling within the typical global range for sedimentary soils (UNSCEAR, 2000). The ^238^U and ^232^Th series are consistently present with ^238^U activity showing considerable spatial variability, and mean values range from 2.46 Bq/kg (Al-Sulaiti et al., 2014) to 213.9 Bq/kg in Dukhan (Al-Sulaiti et al., 2010) (Table 1). Similarly, ^226^Ra (a daughter product of ^238^U) exhibits a wide range from 5 to 350 Bq/kg, though typical mean values are around 17.2 Bq/kg (Al-Sulaiti et al., 2017). ^232^Th activities range between 0.42 and 20 Bq/kg, while ^40^K is a primordial radionuclide and major contributor to natural background radiation with mean concentrations ranged between 10 and 327 Bq/kg. Soil samples from the northwestern part of the Dukhan inshore oil field area show a mean ^226^Ra value of 29.5 Bq/kg with elevated levels up to 342 Bq/kg in specific TENORM affected sites, highlighting the mobility of ^226^Ra in oil field rocks (Al-Sulaiti et al., 2012). The fission product ^137^Cs originating from historical atmospheric nuclear testing is detectable across the Arabian Gulf region. Concentrations of ^137^Cs are generally low with reported mean values ranging from below the minimum detectable activity (MDA) to 7.99 Bq/kg. Other artificial radionuclides like ^90^Sr (mean 3.364 Bq/kg) and plutonium isotopes (^238^Pu, ^239^Pu, ^240^Pu) are present at very low activities, confirming the absence of significant local contamination sources (Amr et al., 2016). Al-Saad et al. (2012) evaluated depleted uranium uptake by sunflower plants under controlled experimental conditions rather than reporting environmental concentrations in natural soils. Following the Gulf wars, several Middle East countries including Kuwait, Iraq, Lebanon, and Egypt showed contamination with depleted uranium, whereas no contamination was observed in Algeria, Isreal, Afghanistan, Oman, Qatar, Iran and Yemen (Besic et al., 2018).

**Table 1:** Concentrations of radionuclides in soil, sediment, seawater, groundwater, building materials and biota from different parts of Qatar

S. No	Location/Study	Number of Samples	Sampling Year	Sample type	Radionuclide	Concentration				References
						Min	Max	Mean	Unit	
1	Across Qatar	129	2008 & 2009	Soil (5–25 cm depth)	^226^Ra	10	350	17.2	Bq/kg	Al-Sulaiti et al. (2017)
					^232^Th	0.42	20	6.38		
					^40^K	10	300	169		
2	Dukhan, Al-Shahaniya	6	2009	Soil (5–15 cm depth)	^238^U	14.97	213.9	54.9	Bq/kg	Al-Sulaiti et al. (2010)
					^232^Th	4.55	13.17	9.4		
					^40^K	111.4	249	204		
					^137^Cs	0.76	20.4	5.9		
3	Al-Shamal, Al-Zubara, Al-Areesh, Fwaret, Ras Lafan, Al-Guwariah, Al-Khawr, Al-Jamaliayah, Dukhan, Al-Shahaniyah and Um Al-Amad	44	2019	Soil (0–16 cm depth)	^226^Ra	5	32	16.6	Bq/kg	Ahmad et al. (2019)
					^232^Th	3	18	10.08		
					^40^K	79	327	200.63		
					^137^Cs	< MDA	47	3.57		
4	Across Qatar	132	2012	Soil (0–10 cm for open soil, 20 cm for vegetable soils, and 30 cm ploughed soil)	^90^Sr	1.0	5.49	3.364	Bq/kg	Amr et al. (2016)
					^137^Cs	0.098	3.993	2.038		
					^238^Pu	< 0.0160	0.0266	0.0195		
					^239^Pu	0.042	0.261	0.150		
					^240^Pu	0.027	0.258	0.103		
5	Across Qatar	129	2008 & 2009	Soil (0–10 cm)	^137^Cs	0.21	15.41	2.15	Bq/kg	Al-Sulaiti et al. (2016a)
6	Dukhan	7	2011	Surface (0–5 cm)	^238^U	15.93 ± 0.30	24.11 ± 0.91	19.17 ± 0.18	Bq/kg	Al-Sulaiti et al. (2014)
					^232^Th	5.65 ± 0.18	13.73 ± 0.54	9.48 ± 0.11		
					^40^K	149.6 ± 7.8	290 ± 15	204.09 ± 4.23		
					^137^Cs	1.65 ± 0.22	19.03 ± 0.89	7.99 ± 0.24		
				Sub Surface (5–25 cm)	^238^U	14.51 ± 0.29	23.63 ± 0.50	18.99 ± 0.14	Bq/kg	
					^232^Th	4.08 ± 0.15	15.60 ± 0.30	9.34 ± 0.08		
					^40^K	128.5 ± 6.8	299 ± 14	203.84 ± 3.94		
					^137^Cs	0.43 ± 0.19	15.41 ± 0.67	6.07 ± 0.16		
		3	2011	Grain Size < 0.5 mm	^238^U	3.53 ± 0.22	28.54 ± 0.45	12.38 ± 0.28	Bq/kg	
					^232^Th	1.12 ± 0.07	8.20 ± 0.21	3.71 ± 0.13		
					^40^K	27.2 ± 2.8	188 ± 10	97.53 ± 4.0		
					^137^Cs	MDA	0.69 ± 0.28	–		
		3	2011	0.5—1 mm	^238^U	3.35	19.22	9.06 ± 0.31	Bq/kg	
					^232^Th	0.69	6.41	2.73 ± 0.12		
					^40^K	22.9	154.0	79.57 ± 3.3		
					^137^Cs	MDA	0.23 ± 0.18	–	Bq/kg	
		3	2011	1—2 mm	^238^U	2.46	16.91	7.97 ± 0.24		
					^232^Th	0.88	5.55	2.66 ± 0.13		
					^40^K	20.9	139.2	67.33 ± 3.2		
					^137^Cs	MDA	0.36 ± 0.17	–		
7	NW Dukhan (inshore oil field area, soil)	32	2011	Soil	^226^Ra	–	342	29.5	Bq/kg	Al-Sulaiti et al. (2012)
					^232^Th	–	–	11		
					^40^K	–	–	320		
	NW Dukhan (TENORM area soil)	2	2011	Soil	^226^Ra	202	342	–	Bq/kg	
8	Dukhan Oil Field (onshore), Qatar	22	2015	Soil	^226^Ra	10.87	45.64	20.05	Bq/kg	Al-Kinani et al. (2015a)
					^232^Th	6.78	46.67	16.43		
					^40^K	112.50	284.20	216.69		
	Dukhan Oil Field (onshore), contaminated soil	3		Contaminated soil	^226^Ra	198.3	289.5	238.17		
					^232^Th	6.7	9.8	8.43		
					^40^K	109.9	120.8	114.4		
	Offshore oil fields (PS field and Al Shaheen field), Qatar	10		Sludge	^226^Ra	394.49	27,884.9	14,577.53		
					^232^Th	30.6	94.1	65.15		
					^40^K	62.8	110.6	97.35		
9	Southern parts of Qatar near Saudi border	21	2012	Soil (50 cm depth)	^238^U	25.008	46.52	31.91	Bq/kg	Al-Kinani et al. (2012a)
					^235^U	0.876	2.470	1.54		
					^226^Ra	25.88	48.99	32.53		
10	South and middle of Qatar (urban areas Saudi border)	21	2012	Soil	^226^Ra	25.01± 0.6	46.52± 1.7	32.02	Bq/kg	Al-Kinani et al. (2012b)
					^232^Th	3.77 ± 0.025	12.37 ± 061	8.88		
					^40^K	125.71 ± 3.5	250.1 ± 7.0	207.34		
					^137^Cs	0.18± 0.01	3.29 ± 0.09	1.04		
11	Qatar (coastal region, oil field area, the rest of Qatar)	5	2011	Soil (5 to 15 cm)	^40^K	–	–	127.1	Bq/kg	Nasir et al. (2012)
					^232^Th	–	–	4.5		
					^238^U	–	–	23.2		
12	EEZ of Qatar	26	2011 & 2012	Sediment	^228^Ra	1.0	6.0	3.1 ± 0.3 (2011); 2.3 ± 0.3 (2012)	Bq/kg	Al-Qaradawi et al. (2015)
					^226^Ra	4.2	19.5	8.5 ± 1.3 (2011); 6.3 ± 0.5 (2012)		
					^238^U	–	–	49.3 ± 3.0 (2011);36.2 ± 1.9 (2012)		
					^232^Th	1	6	–		
					^40^K	11	188	42.1 ± 12.5 (2011); 59.1 ± 16.5 (2012)		
					^137^Cs	0.12	0.66	0.4 ± 0.1 (2011); 0.5 ± 0.1 (2012)		
13	Across Qatar	48	2023	Ground water	^222^Rn	2.71	60.70	20.65	Bq L	Manawi et al. (2023)
14	EEZ of Qatar	15	2011	Seawater	^137^Cs	0.89 ± 0.22	2.31 ± 0.61	1.51 ± 0.46	Bq/m^3^	Al-Qaradawi et al. (2015)
			2012	Seawater	^137^Cs	1.19 ± 0.18	2.17 ± 0.60	1.65 ± 0.32		
15	Qatar	2	2010	Qatari cement	^226^Ra	23	24	23.4 ± 0.6	Bq/kg	Al-Sulaiti et al. (2011)
					^40^K	155	162	158.8 ± 4.3		
					^232^Th	12	13	12.2 ± 0.2		
		4		Sand	^226^Ra	12	14	13.2 ± 0.3	Bq/kg	
					^40^K	216	235	225.5 ± 6.1		
					^232^Th	3	4	3.34 ± 0.05		
		4		Washed sand	^226^Ra	12	14	13.5 ± 0.5	Bq/kg	
					^40^K	210	242	228.2 ± 7.7		
					^232^Th	3	4	3.6 ± 0.2		
		3		White cement	^226^Ra	17	22	18.9 ± 0.5	Bq/kg	
					^40^K	26	87	62.9 ± 22.6		
					^232^Th	4	6	4.9 ± 0.5		
				Clinker	^226^Ra	–	–	23.5 ± 0.3	Bq/kg	
					^40^K	–	–	185 ± 8		
					^232^Th	–	–	9.7 ± 0.2		
					^235^U	–	–	1.4 ± 0.4		
				Kiln feed	^226^Ra	–	–	15.3 ± 0.3	Bq/kg	
					^232^Th	–	–	6.8 ± 0.2		
					^40^K	–	–	139 ± 5		
					^235^U	–	–	0.7 ± 0.2		
				Gypsum	^226^Ra	–	–	11.5 ± 0.2	Bq/kg	
					^40^K	–	–	72 ± 3		
					^232^Th	–	–	2.5 ± 0.1		
					^235^U	–	–	0.4 ± 0.2		
16	Qatar	5	2014–2017	Road building material (clean)	^226^Ra	7	10	8.1	Bq/kg	Al-Kawari and Hushari (2019)
					^232^Th	0.38	6	1.53		
					^40^K	4	25	14.3		
					^137^Cs	0.3	0.8	0.4		
		5		Pure slag from Qatar steel	^226^Ra	260	300	283.4	Bq/kg	
					^232^Th	140	183	166.6		
					^40^K	5.8	20	9.32		
					^137^Cs	0.1	0.8	0.29		
		17		Gabbro	^226^Ra	3.5	19	6.712	Bq/kg	
					^232^Th	0.4	6	1.268		
					^40^K	0.5	25	13.135		
		17		Slag	^226^Ra	225	330	273.1765	Bq/kg	
					^232^Th	13	183	135.1765		
					^40^K	5	30	12.50588		
17	Qatar Steel Yard, Mesaieed Industrial City (Qatar Steel damping area)	10	2015	Steel slag	^226^Ra	257 ± 20	399± 121	324.6	Bq/kg	Al-Kinani et al. (2015b)
					^228^Ra	95 ± 27	128 ± 25	112.3		
					^238^U	120 ± 10	170 ± 41	147.5		
					^232^Th	94 ± 14	127 ± 23	111.7		
					^40^K	7 ± 2	14 ± 3	10		
					^137^Cs	< 0.8	< 1.2	< 1.03		
	Qatar Steel damping area (Asphalt & concrete with mixed slag)	4		Asphalt Mix/Concrete Cube	^226^Ra	75 ± 6	392 ± 121	220 ± 36.6	Bq/kg	
					^228^Ra	23 ± 1	93 ± 27	69.25 ± 8.25		
					^238^U	37 ± 3	170 ± 31	111.75 ± 12.96		
					^232^Th	22 ± 1	93 ± 14	62.25 ± 4.49		
					^40^K	< 7	215 ± 2	67.63 ± 1.97		
					^137^Cs	< 0.6	< 1.4	0.5		
	Mesaieed (Experimental Road – 5 core samples)	5		Asphalt road (top, mid, bottom layers) and concrete	^226^Ra	17 ± 4.4	41 ± 19.6	25.5 ± 5.58	Bq/kg	
					^228^Ra	4.5 ± 0.8	23.8 ± 1	9.75 ± 0.54		
					^235^U	0.8 ± 0.1	2.8 ± 1	1.63 ± 0.32		
					^232^Th	4.3 ± 0.4	22.8 ± 1	9.55 ± 0.38		
					^234^Th	4.7 ± 0.8	41.8 ± 2.6	8.51 ± 0.70		
					^40^K	16 ± 2.8	133 ± 6.5	42.75 ± 1.93		
18	EEZ of Qatar	13	2011–2012	*Pinctada radiata* (Pearl Oyster)	^40^K	–	–	83.7 ± 4.2	Bq/kg	Al-Qaradawi et al. (2015)
					^137^Cs	–	–	<0.08	Bq/kg	
		6		*Lethrinus nebulosus* (Fish)	^40^K	–	–	143 ± 10	Bq/kg	
					^137^Cs	–	–	0.09 ± 0.02	Bq/kg	
		4		*Haliclona* spp. (Sponge)	^40^K	–	–	94.4 ± 4.7	Bq/kg	
					^137^Cs	–	–	0.17 ± 0.03	Bq/kg	
		2		*Halodule univervis* (Seagrass)	^40^K	–	–	131 ± 6.5	Bq/kg	
					^137^Cs	–	–	0.24 ± 0.025	Bq/kg	
		2		*Portunus pelagicus* (Crab)	^40^K	–	–	76.5 ± 3.9	Bq/kg	
					^137^Cs	–	–	< 0.10	Bq/kg	
		1		*Penaeus semisulcatus* (Shrimp)	^40^K			117.9 ± 5.8	Bq/kg	
					^137^Cs			< 0.06	Bq/kg	
		1		*Ophiothrix cf. lineata* (Star fish)	^40^K	–	–	68.5 ± 3.7	Bq/kg	
					^137^Cs	–	–	< 0.25	Bq/kg	
		1		*Dosinia alta* (Clam)	^40^K	–	–	21.3 ± 1.6	Bq/kg	
					^137^Cs	–	–	< 0.25	Bq/kg	
		2		*Dugong dugon* (Sea cow)	^40^K	–	–	55.3 ± 3.1	Bq/kg	
					^137^Cs	–	–	< 0.15	Bq/kg	
		2		*Sargassum benderi* (Algae)	^40^K	–	–	111.6 ± 5.4	Bq/kg	
					^137^Cs	–	–	< 0.04	Bq/kg	
		2		*Avicennia marina* (Mangrove)	^40^K	–	–	21.7 ± 1.2	Bq/kg	
					^137^Cs	–	–	0.42 ± 0.035	Bq/kg	

Vertical profiles from surface (0–5 cm) to subsurface (5–25 cm) layers, particularly in samples from the Dukhan oil field reveal slightly higher activities of ^238^U, ^232^Th, and ^40^K at depth (Al-Sulaiti et al., 2014). This pattern suggests that (i) limited vertical mobility of radionuclides under arid conditions with low leaching potential, (ii) accumulation of fine particles and organic residues in subsurface horizons, and (iii) possible influence from past depositional cycles related to hydrocarbon operations. In contrast, ^137^Cs concentrations decrease exponentially with depth confirming its atmospheric origin and strong affinity for surface soils.

The activity concentrations of natural radionuclides in marine sediments during 2011 and 2012 were generally lower than those in terrestrial soils. Mean concentrations for ^238^U, ^226^Ra and ^228^Ra were reported at 36.2–49.3 Bq/kg, 6.3–8.5 Bq/kg, and 2.3–3.1 Bq/kg, respectively. Mean ^40^K activities were 42.1 Bq/kg (2011) and 59.1 Bq/kg (2012) (Al-Qaradawi et al., 2015). The artificial radionuclide ^137^Cs was detected at very low levels with a maximum value of 0.66 Bq/kg, and many samples below the detection limit, indicating minimal accumulation in seabed sediments (Al-Qaradawi et al., 2015). Sediments near the northern and western coasts-particularly those adjacent to oil operations showed slightly elevated ^238^U and ^226^Ra values but remained below international marine sediment reference levels (UNSCEAR, 2000). The low ^137^Cs content (< 3 Bq/kg) further indicates minimal fallout retention and limited anthropogenic contamination in Qatar’s marine environment. When compared with UNSCEAR reported global averages (^238^U: 33 Bq/kg; ^232^Th: 45 Bq/kg; ^40^K: 420 Bq/kg), radioactivity levels in soils and sediments in the State of Qatar are below global means, confirming a relatively low natural background (Table S1). Marine and coastal sediments exhibit moderate concentrations, with slight variations depending on proximity to industrial zones.

### Groundwater and seawater

A recent nationwide survey of groundwater wells in Qatar measured the concentrations of radon 222 (^222^Rn), a short-lived gaseous decay product of ^238^U. The ^222^Rn levels in groundwater ranged from 2.7 ± 0.2 to 60.7 ± 13.4 Bq/L with a mean of 20.65 Bq/L. Nearly 48% of the analyzed drinking water samples exceeded the US Environmental Protection Agency’s (USEPA, 2011) maximum contamination level of 11.1Bq/L (Manawi et al., 2023). The mean total annual effective dose from ^222^Rn through inhalation and ingestion was estimated at 0.056 mSv/y, remaining below the world health organization’s (WHO, 2004) recommended limit of 0.1 mSv/y. Notably, the contribution from inhalation was found to be higher than that from ingestion, consistent with the volatile nature of radon in confined environments (Manawi et al., 2023). In marine systems, Al-Qaradawi et al. (2015) reported ^137^Cs concentrations in seawater within the EEZ of Qatar that were comparable to background levels in regions unaffected by major nuclear events. Activity concentrations of ^137^Cs in seawater ranged from 0.89 to 2.31 Bq/m^3^, with an average value of 1.51 Bq/m^3^ in 2011, and 1.65 Bq/m^3^ in 2012 (Al-Qaradawi et al., 2015). These values align well with data from the Kuwait marine environment (1.01–1.06 Bq/m^3^) and the broader Indian Ocean, confirming that the detected ^137^Cs originates from historic global fallout rather than local or regional contamination sources.

### Building and construction materials

The radiological characteristics of building materials are a critical determinant of indoor exposure, especially in regions where industrial by-products are commonly incorporated into construction. In Qatar, materials such as cement, sand, concrete, gypsum, and steel slag have been systematically analyzed to assess their natural radionuclide content. Reported concentrations of ^226^Ra, ^232^Th, and ^40^K ranged from 3.5 to 399 Bq/kg, 0.38–183 Bq/kg, and 0.5–242 Bq/kg, respectively (Al-Kinani et al., 2015b; Al-Kuwari & Hushari, 2019; Al-Sulaiti et al., 2011). Al-Sulaiti et al. (2011) further reported the concentrations of ^226^Ra, ^232^Th, ^40^K, and ^235^U in raw materials for cement production in Qatar such as clinker (23.5 ± 0.3, 9.7 ± 0.2, 185 ± 8, and 1.4 ± 0.4 Bq/kg), kiln feed (15.3 ± 0.3, 6.8 ± 0.2, 139 ± 5, and 0.7 ± 0.2 Bq/kg), and gypsum (11.5 ± 0.2, 2.5 ± 0.1, 72 ± 3, and 0.4 ± 0.2 Bq/kg). The radiological assessment of industrial by-products used in road construction materials revealed notable variations. The average activity concentrations of ^40^K, ^232^Th, and ^226^Ra in gabbro samples were 13.135, 1.268 and 6.712 Bq/kg, while corresponding concentrations in slag samples were 12.5, 135.2, and 273.2 Bq/kg (Al-Kuwari & Hushari, 2019). Similarly, Al-Kinani et al. (2015b) reported average activity concentrations of ^226^Ra, ^228^Ra, ^238^U, ^232^Th, ^40^K, and ^137^Cs were 324.6, 112.3, 147.5, 111.7, 10, and < 1.03 Bq/kg, respectively. The elevated concentrations of ^232^Th, and ^226^Ra in steel slag highlight the importance of continuous radiological monitoring before such products reused in construction applications. Regular screening ensures compliance with international safety limits and minimizes long-term indoor radiation exposure.

### Marine organisms

The transfer of radionuclides through the marine food web represents an important pathway for ecological and human populations. In Qatar, the most comprehensive study on radionuclides in marine biota was conducted by Al-Qaradawi et al. (2015), encompassing a diverse range of organisms collected from the EEZ between 2011 and 2012. Species analyzed included fish (*Lethrinus nebulosus*), mollusks (*Dosinia alta*), crustaceans (*Penaeus semisulcatus*), macroalgae (*Sargassum benderi*), sponge (*Haliclona* spp.), seagrass (*Halodule univervis*), mangrove (*Avicennia marina*) and the dugong (*Dugong dugon*). The findings provide a crucial baseline for understanding bioaccumulation patterns in the arid marine environment of the Arabian Gulf. ^40^K was detected in all organisms, reflecting its physiological role as an essential element in cellular metabolism. Activity concentrations varied across species, ranging from 21.3 ± 1.6 Bq/kg in *Dosinia alta* to 143 ± 10 Bq/kg in *Lethrinus nebulosus*, values that are consistent with the global averages for marine biota and pose no radiological concern. The artificial radionuclide ^137^Cs was either below the minimum detectable activity (MDA) or found in trace levels, ranging from 0.04 Bq/kg in *Sargassum benderi* to 0.42 ± 0.035 Bq/kg in *Avicennia marina*. Contamination factor analysis revealed the following order of ^137^Cs concentration: mangroves > seagrass > sponges > fish, suggesting higher affinity of sediment-associated biota for cesium. These results indicate low ^137^Cs contamination in the investigated marine biota. The available studies on marine biota primarily report radionuclide activity concentrations in selected organisms. Although these data provide valuable information on radionuclide occurrence and bioaccumulation, they are insufficient to perform a comprehensive ecological risk assessment. Therefore, the present review summarizes the available concentration data but does not evaluate potential radiological risks to aquatic organisms and ecosystem health.

## Sources of environmental radionuclides in Qatar

The occurrence of radionuclides in Qatar is primarily governed by natural geological processes, with additional contributions from anthropogenic and transboundary sources (Al-Sulaiti et al., 2010). Understanding both natural and anthropogenic origins is critical for assessing radionuclide distribution patterns and associated radiological risks across various environmental matrices.

### Natural sources

The primary source of environmental radioactivity in Qatar is naturally occurring radioactive material (NORM), derived from the decay series of ^238^U and ^232^Th and the ubiquitous ^40^K. These radionuclides are intrinsic components of the bedrock in Qatar, predominantly the Eocene Rus and Dammam formations composed of carbonate and evaporitic lithologies. Therefore, lithological characteristics exert a primary control on radionuclide distribution. Carbonate-rich rocks tend to immobilize uranium and radium through co-precipitation with calcium carbonate, whereas clay-rich or phosphatic strata often host higher thorium and potassium isotopes. Soils and sediments derived from these geological formations exhibit a wide range of activity concentrations, reflecting mineralogical heterogeneity and geochemical conditions. Fine-grained and clay-rich soils exhibit greater adsorption capacity for ^238^U, ^232^Th, and ^226^Ra due to their high specific surface area and cation exchange capacity. Ahmad et al. (2019) studied the vertical distribution of radionuclides in different parts of Qatar (Dukhan, Al-Shamal, Al-Areesh, Al-Zubara, Fuwairit, Al-Guwariah, Ras Lafan, Al-Jamaliayah, Al-Shahaniyah, Al-Khor and Um Al-) have reported average concentrations of 10.08 Bq/kg for ^232^Th, 16.6 Bq/kg for ^226^Ra, 200.63 Bq/kg for ^40^K and 3.57 Bq/kg for ^137^Cs. The hyper-arid climate and unique geochemical conditions, particularly in sabkha (coastal salt flat) environments, promote radionuclide retention through evaporative concentration and adsorption onto carbonate and sulfate minerals (Al-Sulaiti et al., 2014). High evaporation rates increase salinity and ionic strength, enhancing radium and uranium fixation, while minimal rainfall restricts leaching, leading to surface accumulation rather than vertical migration.

Dust storms and wind erosion are key natural mechanisms for radionuclide redistribution across the Qatar peninsula. Originating from both local (sabkha and desert), and regional (northern Saudi Arabia and Iraq) sources, these storms transport fine mineral particles enriched in adsorbed radionuclides. Concentrations of ^238^U, ^232^Th, and ^40^K in airborne dust have been reported to be two to three times higher than in local soils (Ababneh et al., 2018). The deposition of this dust contributes to seasonal enrichment of radionuclides in surface soils and sediments, particularly in northern and coastal Qatar. Airborne radionuclides can also attach to aerosol particles and settle in marine environments, slightly elevating uranium and potassium levels in seawater and coastal sediments. Although the total radiological impact remains low, dust storms play a critical role in the spatial redistribution of radionuclides across Qatar and the wider Arabian Gulf (Nabavi et al., 2023).

### Anthropogenic sources

Despite the dominance of natural background radiation, several human activities have introduced or intensified radionuclide presence in localized areas. The hydrocarbon represents the most significant source of TENORM in Qatar. Oil and gas operations, particularly in the northwestern Dukhan field, generate scale deposits, drill muds, and produced water containing elevated levels of radium isotopes (^226^Ra, ^228^Ra). Soil samples from these areas have shown ^226^Ra concentrations up to 342 Bq/kg, which is nearly tenfold above the global average of 33 Bq/kg, and creating localized zones of TENORM contamination (Al-Sulaiti et al., 2012). Locally manufactured building materials such as cement, steel slag, and concrete contain measurable concentrations of NORM. Mean activity concentrations in cement and slag-based materials were reported as 12–13 Bq/kg for ^232^Th, 155–162 Bq/kg for ^40^K, and 23–24 Bq/kg for ^226^Ra, attributed to the incorporation of industrial by-products during the smelting process. These findings highlight an indirect TENORM pathway into urban infrastructure (Al-Sulaiti et al., 2011).

### Artificial and transboundary inputs

Trace levels of artificial radionuclide ^137^Cs have been detected in surface soils and sediments, ranging from 0.21 up to 15.41 Bq/kg. The presence of ^137^Cs primarily reflects global atmospheric fallout from mid-twentieth century nuclear weapons testing and, to a lesser extent, long-range transport from historic nuclear accidents such as Chernobyl and Fukushima (Al-Sulaiti et al., 2016a; Nabavi et al., 2023). Al-Sulaiti et al. (2010) reported mean ^137^Cs concentrations of 5.8 ± 5.6 Bq/kg in surface soils (5–15 cm), values typical for regions unaffected by direct contamination. Although radiologically insignificant, ^137^Cs serves as a useful tracer for soil erosion and sedimentation studies (Ahmad et al., 2019). Regional atmospheric and marine transport processes further contribute to minor radionuclide inputs. Prevailing northwesterly ‘Shamal’ winds can carry airborne radionuclides, especially ^137^Cs from distant sources such as Iraq, Iran, or the Levant. Similarly, the semi-enclosed Arabian Gulf circulation allows limited redistribution of radionuclides through marine currents, especially near the EEZ boundaries (Perianez, 2021). Though no significant anthropogenic contamination has been reported in coastal or offshore waters of Qatar, transboundary transport highlights the importance of regional cooperation for radiological monitoring (Al-Dousari et al., 2022; Nesterov et al., 2023).

### Secondary and industrial water sources

Like many countries in the Arabian Gulf, Qatar relies heavily on seawater desalination for its potable water supply. Intake waters and brine reject streams in the desalination plant may contain trace levels of ^226^Ra and ^228^Ra. Similarly, industrial wastewater discharges from petrochemical facilities and power plants may release minor quantities of radionuclides into nearshore environments (Ali & Kakosimos, 2023; Babatunde et al., 2019; Maderich et al., 2023). The overall distribution of radionuclides in Qatar is governed by strong geochemical controls. The high calcium carbonate-rich soils, alkaline pH, and low organic matter favor adsorption over mobility, leading to radionuclide accumulation in fine-grained sediments, which act as long-term sinks (Al-Sulaiti et al., 2014). In contrast, biological uptake and trophic transfer remain limited. The bioaccumulation of ^40^K in marine biota is part of normal physiological processes, while uranium and thorium show negligible assimilation and weak food web transfer (Al-Qaradawi et al., 2015). Consequently, radionuclide cycling in Qatar is primarily controlled by geological, climatic, and industrial factors rather than biological processes.

## Radiological risk assessment

The radiological risk assessment in this study was restricted to solid environmental and industrial matrices for which the activity concentrations of ^226^Ra, ^232^Th, and ^40^K were available for calculation of standardized radiological risk indices. Accordingly, Radium equivalent activity (Ra_eq_), External hazard index (H_ex_), Internal hazard index (H_in_), Annual gonadal dose equivalent (AGDE), Annual effective dose equivalent (AEDE), and Excess lifetime cancer risk (ELCR) were calculated only for solid materials (soil, sediment and industrial matrices), and were not calculated for groundwater, seawater, and biological matrices (Alshahrani et al., 2025). For these non-solid matrices, the review instead summarizes the reported radionuclide concentrations and their observed distributions.

 The sludge samples represent technologically enhanced naturally occurring radioactive materials (TENORM) collected from oil field separation tanks during hydrocarbon production. These industrial residues contain concentrated naturally occurring radionuclides resulting from production processes and therefore represent localized occupational exposure scenarios rather than typical environmental background conditions. Consequently, the elevated radiological indices reported for sludge should be interpreted separately from those of natural environmental matrices and should not be considered representative of public environmental exposure in Qatar.

### ***Radium equivalent activity (Ra***_***eq***_***)***

The radium equivalent activity (Ra_eq_) is a standardized radiological index used to assess the total gamma radiation hazard of a material by combining the activity concentrations of ^226^Ra, ^232^Th, and ^40^K into a single representative value. It is calculated as follows:

$${Ra}_{eq}(Bq/kg)={A}_{Ra}+1.43*{A}_{Th}+0.077*{A}_{K}$$

where, A_Ra_, A_Th_, and A_K_ are the activity concentrations (in Bq/kg) of ^226^Ra, ^232^Th, and ^40^K, respectively. The coefficients account for the relative gamma dose contributions of each radionuclide. According to UNSCEAR (2000), the recommended safety limit for Ra_eq_ is 370 Bq/kg, which corresponds to an annual effective dose of 1 mSv for the public.

**Fig. 4:**
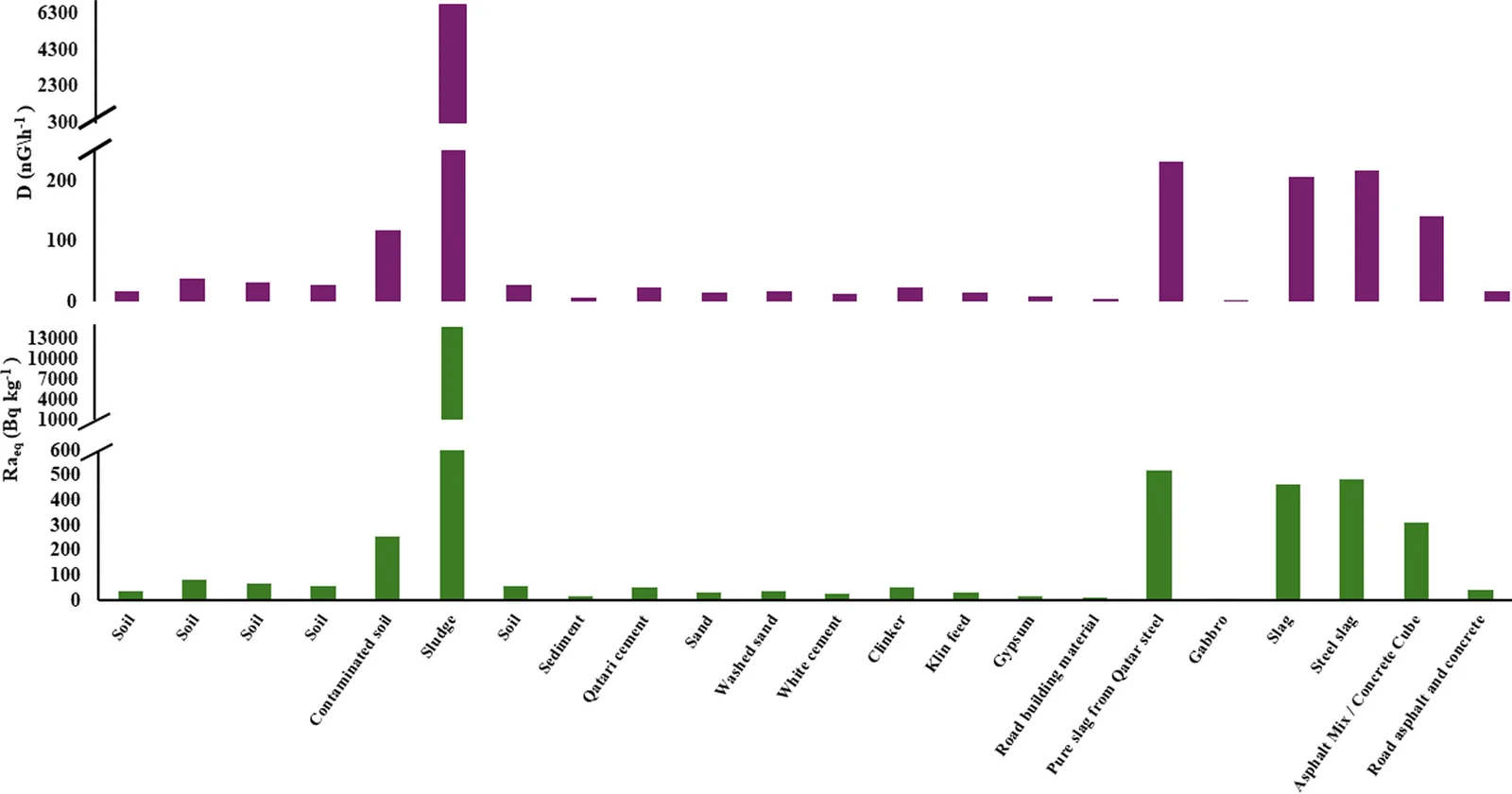
Distribution of radium equivalent activity (Ra_eq_) and absorbed dose rate in air (D) among different environmental sampling matrices in Qatar. Elevated values observed for sludge represent TENORM originating from oil field separation facilities and illustrate localized industrial radiological hazards rather than natural environmental background radiation or routine public exposure

Across all environmental matrices analyzed, Ra_eq_ values ranged from 9.54 to 14,678.19 Bq/kg (Fig. 4, Table S2) with an overall average of approximately 790.11 Bq/kg. However, this average is strongly influenced by the exceptionally high Ra_eq value observed in industrial sludge, which represents a TENORM residue from oil-field operations. Excluding this extreme outlier, the average Ra_eq for the remaining environmental matrices decreases to 128.8 Bq/kg, indicating that most natural soils, marine sediments, and conventional building materials such as cement, sand, and gypsum exhibited Ra_eq_ values well below the recommended safety threshold (< 370 Bq/kg), suggesting negligible radiological risk (Table S3). In contrast, industrial residues such as sludge (14678.19 Bq/kg), and slag (467–522 Bq/kg) exhibited exceptionally high Ra_eq_ values. It should be emphasized that the sludge samples were collected from oil field separation tanks and represent TENORM generated during hydrocarbon extraction and processing activities rather than naturally occurring environmental soils. Consequently, these values reflect localized industrial contamination and potential occupational exposure rather than public exposure. The exceptionally high Ra_eq_ observed in sludge highlights the importance of appropriate handling, storage, transportation, and disposal of TENORM wastes to minimize occupational and environmental risks. Overall, natural soils, sediments, cement, sand and gypsum exhibit comparatively low Raₑ_q_ values, reflecting lower radionuclide concentrations. In contrast, sludge showed the highest Raₑ_q_ value, indicating substantial accumulation of natural radionuclides and a greater radiological contribution. Industrial by-products such as steel slag and asphalt mixtures also demonstrated relatively elevated values, likely due to mineral concentration and processing related enrichment. The observed variability in Ra_eq_ values can be attributed to differences in geological composition, industrial activities, and material processing.

### Absorbed dose rate in air (D)

The external gamma radiation hazard from environmental radionuclides can be quantified by the absorbed dose rate in air (D) at 1 m above ground level, which reflects the contributions of ^226^Ra, ^232^Th, and ^40^K present in soils, sediments, building materials, and industrial by-products. This parameter provides a preliminary estimate of potential external exposure, though the actual dose received by humans depends on factors such as outdoor occupancy time, exposure patterns, and shielding provided by buildings or other materials. The absorbed dose rate is calculated using the following equation:

$$D (nGy/h)=0.462{A}_{Ra}+0.604{A}_{Th}+0.042{A}_{K}$$

In Qatar, the calculated absorbed gamma dose rates (D) varied considerably across different environmental matrices, ranging from 4.42 nGy/h in gabbro samples to 6778.26 nGy/h in sludge (Fig. 4, Table S2). Natural soils exhibited D values ranging from 18.9 to 39.4 nGy/h, which are generally below the global average of 59 nGy/h reported by UNSCEAR (2000). These findings indicate that under typical environmental conditions, gamma radiation levels in most terrestrial and marine matrices in Qatar pose a low external radiological hazard. However, elevated values observed in certain industrial residues, particularly sludge, indicate localized zones of enhanced external exposure that may require monitoring and appropriate mitigation measures. The results demonstrate clear differences between conventional construction materials and industrial by-products, primarily due to variations in radionuclide concentrations. The exceptionally high absorbed dose rates observed in sludge represent localized TENORM residues associated with oil field operations and should not be interpreted as representative of natural environmental radiation levels in Qatar. In contrast, washed sand, white cement, gypsum, and gabbro showed comparatively low radiation levels. Overall, the results indicate that industrial residues contribute to more environmental radiation exposure than common building materials.

### Annual effective dose equivalent (AEDE)

The annual effective dose equivalent (AEDE) is an important parameter for assessing human health risk associated with long-term exposure to environmental radioactivity. It is derived from the absorbed dose rate in air (D) and accounts for both the biological effectiveness of gamma radiation and human exposure patterns, following the methodology recommended by UNSCEAR (2000). Two important conversion factors are applied in its calculation: (i) a dose conversion coefficient of 0.7 Sv/Gy, which converts the absorbed dose in air to the effective dose received by adults, and (ii) an outdoor occupancy factor of 0.2, representing the average fraction of time an individual spends outdoors. The AEDE is calculated using the following equation:

$$\mathrm{A}\mathrm{E}\mathrm{D}\mathrm{E} (\mathrm{m}\mathrm{S}\mathrm{v}/\mathrm{y})=\mathrm{D}(nGy/h)*8760\mathrm{h}*0.2*0.7*{10}^{-6}$$

**Fig. 5:**
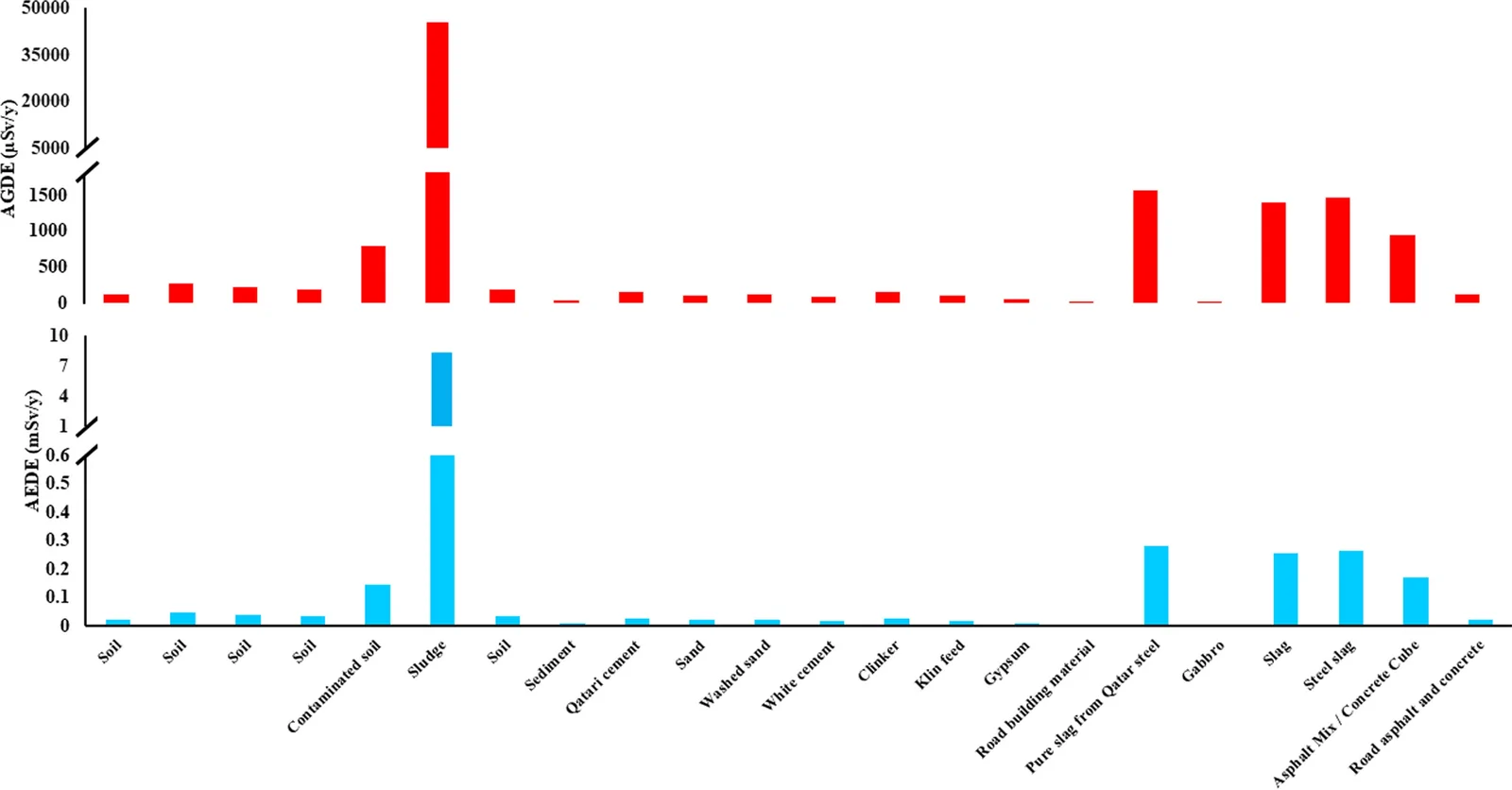
Annual effective dose equivalent (AEDE) and annual gonadal dose equivalent (AGDE) across different environmental sampling matrices in Qatar. The elevated dose values associated with sludge reflect TENORM residues generated during hydrocarbon production and represent localized occupational exposure scenarios rather than typical environmental conditions

The AEDE values in natural soils in Qatar ranged from 0.023 to 0.048 mSv/y, which are well below the global average of 0.07 mSv/y for outdoor terrestrial radiation exposure (Fig. 5, Table S2). These results indicate minimal radiological risk to the general population under normal environmental conditions. In contrast, the AEDE value for sludge reached 8.313 mSv/y, exceeding the recommended public dose limit of 1 mSv/y nearly seven-fold. Such elevated AEDE values calculated for sludge correspond to occupational exposure scenarios associated with TENORM management rather than routine public exposure. Most environmental matrices and conventional construction materials exhibited AEDE values below the recommended safety limit, indicating their radiological suitability for environmental and construction-related applications. However, contaminated soils and certain industrial by-products, such as slag and asphalt mixtures showed moderately elevated AEDE values, reflecting localized radionuclide enrichment. The exceptionally high AEDE observed in sludge is attributed to the accumulation of radioactive minerals and industrial residues during hydrocarbon extraction and processing activities. These findings suggest that while most materials analyzed in Qatar are radiologically safe, industrial waste materials (especially sludge) require continuous monitoring, proper waste management, and appropriate regulatory oversight to minimize potential long-term exposure risks.

### Annual gonadal dose equivalent (AGDE)

The annual gonadal dose equivalent (AGDE) is a dosimetric parameter used to quantify the radiation dose received by the gonads, providing an estimate of potential genetic risk to the population. In addition to the gonads, active bone marrow is considered a highly radiosensitive tissue due to its susceptibility to stochastic effects such as leukemia. Therefore, AGDE serves as an important indicator for evaluating both heritable effects and potential bone marrow irradiation resulting from environmental radionuclide exposure. AGDE was calculated based on the activity concentrations of ^226^Ra, ^232^Th, and ^40^K using the following equation:

$$AGDE (\mu Sv/y)=3.09{A}_{Ra}+{4.18A}_{Th}+0.314{A}_{K}$$

The calculated AGDE values ranged from 30.16 µSv/y in gabbro samples to 45,347.46 µSv/y in sludge (Fig. 5, Table S2). For most natural soils, marine sediments, and conventional construction materials, AGDE values ranged between 100 and 250 µSv/y, remaining below the suggested natural background reference limit of 300 µSv/y. These results indicate relatively low radiological impact and minimal genetic risk under normal environmental conditions. The elevated AGDE values reported for sludge reflect concentration radionuclides in industrial TENORM residues rather than naturally occurring environmental matrices. Among all analyzed matrices, sludge recorded the highest AGDE values, reflecting substantial enrichment of ^226^Ra, ^232^Th, and ^40^K, likely associated with radionuclide accumulation during hydrocarbon extraction and processing. Similarly, slag-related materials also showed elevated AGDE values compared with conventional construction materials, indicating moderate radiological concern. These findings emphasize the need for restricted human access, proper handling, and controlled disposal of industrial residues to minimize potential genetic and stochastic health risks. Overall, soils, sand, gypsum, cement, and concrete materials exhibited comparatively low AGDE values, supporting their radiological suitability for environmental and construction applications, whereas industrial residues require continued monitoring and effective management strategies.

### ***External and internal hazard indices (H***_***ex***_*** and H***_***in***_***)***

The external hazard index (H_ex_) and internal hazard index (H_in_) are widely used radiological parameters for evaluating potential hazards associated with external gamma radiation exposure and internal exposure due to radon and its progeny, respectively. These indices are designed to ensure that radiation exposure levels remain within internationally recommended safety limits. The indices are calculated using the following equations:

$${H}_{ex}=\frac{{A}_{Ra}}{370}+\frac{{A}_{Th}}{259}+\frac{{A}_{K}}{4810}$$

$${H}_{in}=\frac{{A}_{Ra}}{185}+\frac{{A}_{Th}}{259}+\frac{{A}_{K}}{4810}$$

**Fig. 6:**
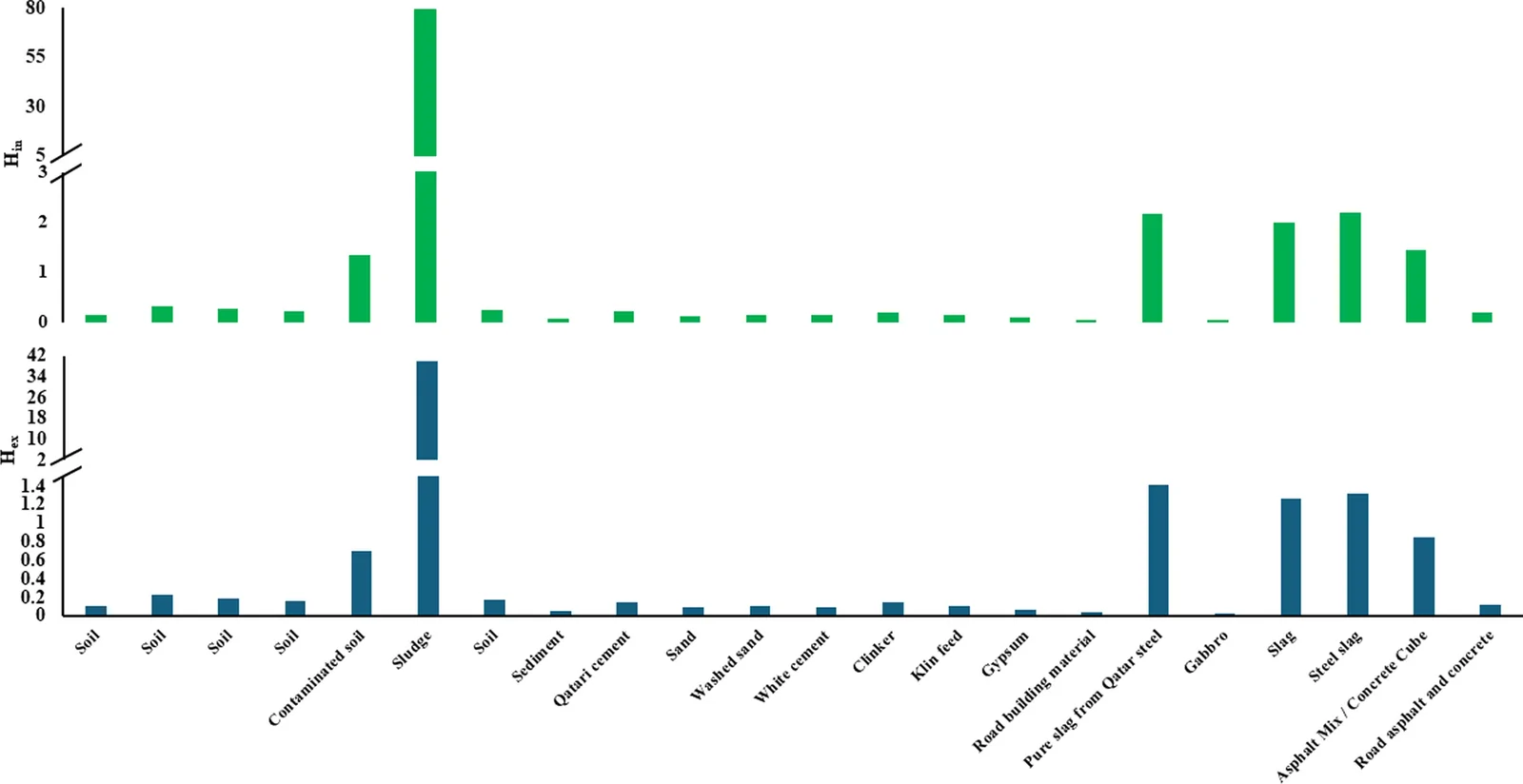
Comparison of external and internal hazard indices among different environmental sampling matrices in Qatar. Hazard indices exceeding the recommended safety limit are primarily associated with industrial TENORM residues from oil field operations and should be interpreted separately from natural environmental matrices and conventional construction materials

For a material to be considered radiologically safe, both H_ex_ and H_in_ should be less than unity (< 1). In Qatar, natural soils, marine sediments, and conventional construction materials exhibited low hazard index values, ranging from 0.03 to 0.22 for H_ex_ and 0.05 to 0.31 for H_in_ (Fig. 6, Table S2). These values indicate minimal radiological risk and confirm their suitability for environmental and construction applications. In contrast, industrial residues such as sludge (H_ex_ = 39.67; H_in_ = 79.07) and slag (H_ex_ = 1.26–1.41; H_in_ = 2–2.18) significantly exceeded the recommended safety threshold. These elevated external and internal hazard indices associated with sludge primarily indicate occupational radiological hazards during handling and disposal of TENORM wastes and are not representative of environmental background conditions. The exceptionally high H_in_ values observed in sludge indicate an increased potential for internal exposure through radon inhalation, while the elevated H_ex_ values reflect substantial external gamma radiation hazards. These findings suggest that industrial waste materials may pose considerable long-term radiological risks and therefore require stringent regulatory control, proper storage, and specialized disposal strategies. Overall, the results demonstrate that most used building materials in Qatar are radiologically safe, whereas certain industrial by-products and waste residues pose significant radiological concerns that warrant continuous monitoring and careful management.

### Excess lifetime cancer risk (ELCR)

The excess lifetime cancer risk (ELCR) is a quantitative parameter used to estimate the probability of an individual developing cancer over a lifetime due to exposure to a carcinogenic agent. In the context of environmental radioactivity, ELCR quantifies the additional risk attributable to prolonged exposure to natural or anthropogenic radionuclides beyond the baseline incidence of cancer. The ELCR was calculated using the following equation:

ELCR=AEDE(Sv/y)∗DL∗RF

where AEDE is the annual effective dose equivalent (in Sv/y), DL is the average duration of life, taken as 70 years, and RF is the fatal cancer risk factor, taken as 0.05 Sv^−1^, as recommended by the international commission on radiological protection (ICRP Publication 103, 2007) (Streffer, 2007). The product of these parameters provides an estimate of the additional probability of developing fatal cancer over an average lifetime due to radiation exposure.

**Fig. 7:**
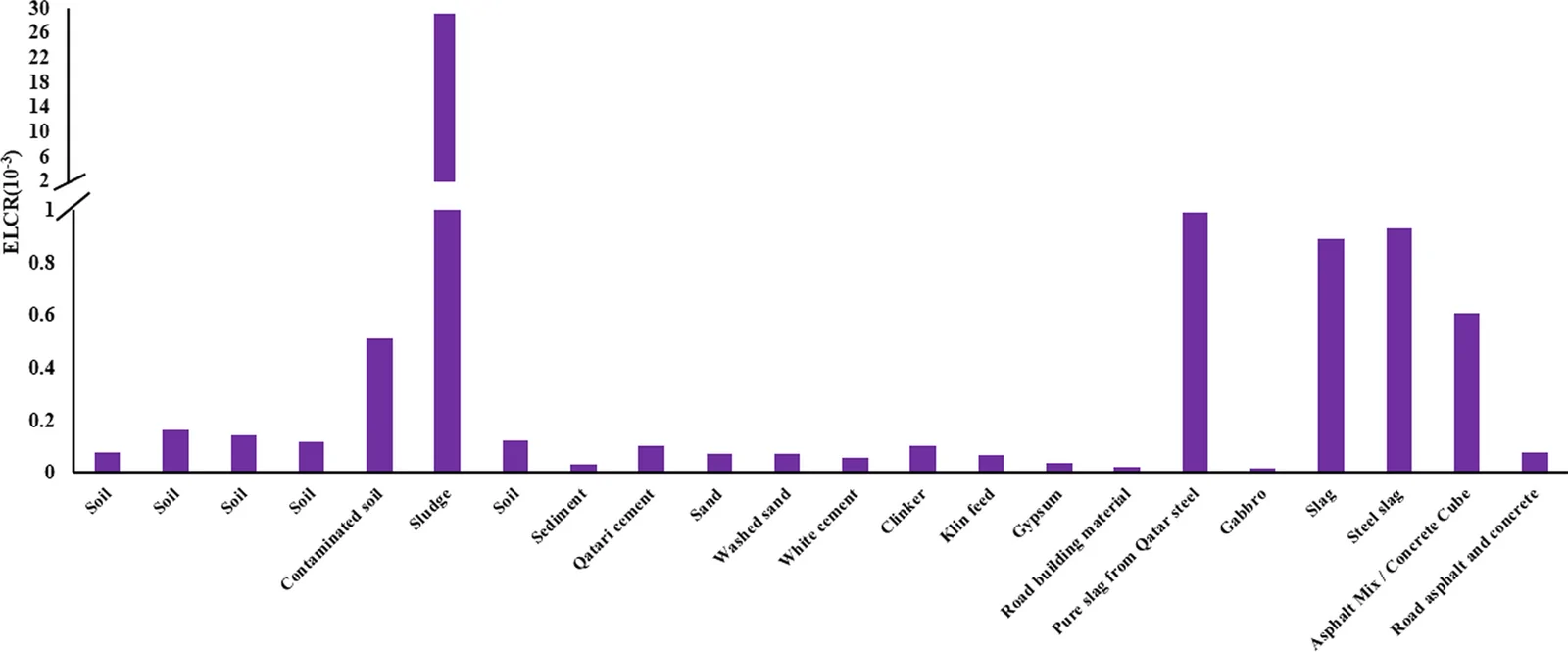
Variation in excess lifetime cancer risk (ELCR) across different environmental sampling matrices in Qatar. Elevated ELCR values for sludge represent localized industrial TENORM residues associated with hydrocarbon production and are indicative of potential occupational exposure rather than the radiological risk to the public under normal environmental conditions

The calculated ELCR values in Qatar varied from 0.019 × 10^–3^ for gabbro samples to 29.095 × 10^–3^ for sludge (Fig. 7, Table S2). The mean ELCR for natural soil samples was around 0.12 × 10^–3^, which is lower than the global average of 0.29 × 10^–3^ reported by UNSCEAR (2000), indicating negligible radiological risk to the general population under normal environmental conditions. However, industrial residues such as sludge and slag samples displayed significantly elevated ECLR values. The high ELCR estimated for sludge reflects a conservative assessment of localized industrial TENORM residues rather than the lifetime cancer risk for the general population living under normal environmental conditions. The elevated risk associated with industrial sludge reflects significant radionuclide accumulation, particularly radium isotopes, likely associated with hydrocarbon extraction and processing activities. Moderately elevated ELCR values were also observed in contaminated soils, slag, steel slag, and asphalt-mix concrete cubes suggesting localized radiological concerns. In contrast, most natural soils, marine sediments, sand, gypsum, clinker, and white cement exhibited comparatively low ELCR values indicating satisfactory radiological safety and minimal cancer risk.

Overall, natural soils, sediments, and conventional building materials in Qatar pose minimal radiological risk. In contrast, industrial by-products and oil-field residues exhibit elevated radionuclide concentrations and associated risk indices, consistent with regional observations in the Arabian Gulf (Alotaibi et al., 2024), where oil-industry wastes accumulate radium isotopes in sludge and scale. Although this review identified extremely high radiological indices for sludge, these values should be interpreted within the context of TENORM generated by the hydrocarbon industry in Qatar. Oil field sludge and production scale are well recognized repositories of naturally occurring radionuclides that become concentrated during oil and gas extraction and processing. Therefore, these materials represent localized industrial waste requiring appropriate occupational radiation protection, waste management, storage, transport, and disposal practices. They should not be interpreted as indicative of elevated environmental background radiation or radiological exposure to the public. In contrast, the radiological indices for natural soils, sediments, groundwater, seawater, and conventional construction materials remain well within internationally accepted safety limits, indicating minimal radiological risk under normal environmental conditions.

## Management strategies and policy framework

Effective management of environmental radionuclides in Qatar requires a comprehensive framework integrating scientific monitoring, regulatory oversight, and proactive policy implementation. This requirement is further amplified by the presence of active nuclear power plants and other facilities in neighboring countries and planned new installations, which introduce potential transboundary radiological risks. The proposed framework addresses both local sources such as NORM and TENORM from oil and gas extraction, desalination, and industrial activities, and external threats, ensuring alignment with Qatar National Vision 2030. Radioactivity monitoring must be expanded beyond baseline mapping of natural radionuclides to include real-time atmospheric and marine surveillance networks. These systems should be specifically calibrated to detect artificial radionuclides that could result from an incident at regional nuclear facilities. The integration of this data into a centralized national database, linked with the International Atomic Energy Agency’s International Radiation Monitoring Information System (IRMIS) is critical for early warning and rapid response capabilities.

While the existing radiation protection law in Qatar provides a foundational regulatory framework, updates are necessary to address contemporary challenges. To address external risks, pre-emptive public health guidelines must be developed to manage potential fallout, establishing actionable thresholds for radionuclide concentrations on imported foodstuffs and local agricultural products. Furthermore, regional collaboration within the ROPME Sea area should be strengthened through harmonized nuclear safety protocols and formal bilateral agreements for immediate information sharing and technical assistance in the event of a nuclear emergency. The integrated management approach ensures robust radiological protection, aligns with international standards, and safeguards both public health and the environment against current and emerging radiological risks.

## Research gaps and future directions

Although considerable progress has been made in understanding the occurrence and distribution of radionuclides in Qatar, several important knowledge gaps remain. Most previous studies have focused on measuring radionuclide activity concentrations in individual environmental matrices or specific industrial settings, whereas comprehensive nationwide assessments integrating multiple environmental compartments remain limited. Furthermore, the rapid expansion of industrial activities, urban development, desalination, and oil and gas production highlights the need for continuous monitoring and improved radiological risk assessment strategies. A major priority for future research is the establishment of a long-term nationwide environmental radioactivity monitoring program encompassing terrestrial, freshwater, coastal, and marine ecosystems. Such a program should include periodic sampling of soils, sediments, groundwater, seawater, atmospheric dust, construction materials, agricultural products, and marine organisms to identify temporal trends, detect emerging contamination, and evaluate the effectiveness of environmental protection measures. Continuous monitoring is particularly important in rapidly developing industrial regions and areas influenced by hydrocarbon production.

Future investigations should also evaluate the potential influence of climate change on radionuclide mobility and redistribution. Increasing temperatures, prolonged droughts, changing precipitation patterns, sea level rise, coastal erosion, and more frequent dust storms may alter the transport, resuspension, and bioavailability of radionuclides in arid environments. Understanding these processes will improve predictions of future exposure scenarios under changing climatic conditions. Greater emphasis should be placed on isotopic fingerprinting techniques to distinguish between naturally occurring radionuclides, technologically enhanced naturally occurring radioactive materials (TENORM), and anthropogenic radionuclides. High-precision isotope ratio measurements using advanced mass spectrometric techniques can improve source attribution and help differentiate radionuclides originating from geological formations, oil and gas production, phosphate fertilizers, industrial activities, and global atmospheric fallout. Such approaches would strengthen environmental forensic investigations and facilitate more effective management of contamination sources.

Considering extensive hydrocarbon industry in the State of Qatar, further research should focus on the comprehensive characterization of TENORM generated during oil and gas exploration, production, refining, and waste management. Detailed investigations are needed to quantify radionuclide accumulation in production sludge, scales, produced water, drilling wastes, refinery residues, and associated industrial by-products. These studies should include assessments of radionuclide speciation, leachability, waste classification, occupational exposure, and long-term disposal strategies to support sustainable TENORM management. The development of radionuclide transport models represents another important research priority. Numerical models integrating atmospheric dispersion, groundwater transport, sediment dynamics, and marine circulation would improve understanding of radionuclide migration under both routine and accidental release scenarios. Such models can support environmental impact assessments, emergency preparedness, and decision-making for environmental management. Another significant research gap is the limited availability of transfer coefficients for radionuclides under arid environmental conditions. Most existing transfer parameters used in environmental risk assessments have been derived from temperate ecosystems and may not adequately represent radionuclide behavior in desert soils, high salinity coastal environments, and hyper-arid climate in Qatar. Site specific transfer factors describing radionuclide movement from soil to plants, sediments to marine organisms, and water to aquatic biota would substantially improve the accuracy of human and ecological dose assessments. Although radionuclide concentrations have been reported in several marine organisms, formal ecological (biota) radiological risk assessments remain largely absent. Future studies should evaluate radionuclide bioaccumulation, trophic transfer, biomagnification, and potential effects on marine biodiversity using internationally recognized assessment frameworks, such as the IAEA’s ERICA (Environmental Risk from Ionising Contaminants: Assessment and Management) approach. Such investigations would provide a more comprehensive understanding of environmental impacts beyond human health risk assessments.

Emerging digital technologies provide significant opportunities to modernize environmental radioactivity surveillance. The integration of artificial intelligence (AI), machine learning, Geographic Information Systems (GIS), remote sensing, and Internet of Things (IoT)-based sensor networks can facilitate near real-time monitoring, spatial prediction of radionuclide distributions, anomaly detection, and automated risk mapping. These technologies can improve the efficiency of environmental monitoring programs, optimize sampling strategies, and support evidence-based environmental management. Given that the Arabian Gulf is a shared marine ecosystem, enhanced regional cooperation through the Regional Organization for the Protection of the Marine Environment (ROPME) should be encouraged. Establishing a coordinated radionuclide monitoring network among Gulf countries would facilitate standardized sampling protocols, data sharing, inter-laboratory comparisons, and early warning systems for transboundary radiological contamination. Such collaboration would improve regional preparedness and contribute to the long-term protection of the Gulf marine environment. Future research should also actively participate in IAEA coordinated research programs and international intercomparison exercises to harmonize analytical methodologies, quality assurance procedures, and radiological risk assessment approaches. Participation in international initiatives would strengthen analytical capabilities, improve data comparability, and ensure that environmental radionuclides assessments in Qatar remain aligned with global base practices.

From a policy perspective, there is a need to further strengthen regulatory framework of Qatar for environmental radioactivity management. Future efforts should focus on developing comprehensive national guidelines for monitoring naturally occurring radionuclides and TENORM, establishing radionuclide baseline inventories, implementing standardized protocols for industrial waste characterization, and defining clear criteria for the storage, transport, disposal, and potential reuse of TENORM-containing materials. Regular environmental surveillance around oil and gas facilities, industrial zones, and waste disposal sites should be incorporated into national environmental monitoring programs. Furthermore, closer alignment with the recommendations of the International Atomic Energy Agency (IAEA), International Commission on Radiological Protection (ICRP), and United Nations Scientific Committee on the Effects of Atomic Radiation (UNSCEAR) would further strengthen the capacity of Qatar for environmental radiation protection and sustainable industrial development. Overall, future research should adopt an interdisciplinary approach combining environmental radioactivity, geochemistry, atmospheric science, marine ecology, remote sensing, numerical modelling, and data science to improve understanding of radionuclide behavior in arid environments. Such integrated efforts will provide a robust scientific foundation for evidence-based policy development, sustainable environmental management, and long-term protection of human health and ecosystems in Qatar and the wider Arabian Gulf region.

## Conclusions

This study provides a systematic overview of environmental radionuclides in Qatar, highlighting the distribution, sources, and associated radiological risks across different matrices. Natural radionuclides dominate the environmental radioactivity profile with concentrations largely controlled by geology, soil characteristics, and climatic factors. The artificial radionuclides are negligible, reflecting the absence of significant local nuclear contamination. Radiological risk indices confirm that most soils, sediments, and building materials pose minimal health risks, whereas industrial by-products, oil-field residues, and waste materials exhibit elevated radionuclide activity and potential localized hazards, highlighting careful management and regulatory oversight. Dust storms, arid conditions, and limited water mobility influence radionuclide redistribution, whereas minor transboundary contributions from regional atmospheric and marine transport are detectable but low.

The limitation of this study is that radiological risk assessments were confirmed to human exposure using established radiological indices. Although radionuclide concentrations reported in marine organisms were compiled and discussed, a quantitative ecological risk assessment was beyond the scope of this study because of the limited availability of species-specific transfer parameters and dose assessment data. Similarly, the policy discussion focuses on future recommendations rather than providing a comprehensive evaluation of existing regulatory framework in Qatar. Overall, this study demonstrates that natural environmental matrices in Qatar, including soils, sediments, and conventional construction materials exhibit low levels of radiological risk and generally comply with international safety recommendations. In contrast, industrial residues associated with hydrocarbon production, particularly oil field sludge and production scale contain elevated concentrations of TENORM and consequently exhibit substantially higher radiological hazard indices. These findings primarily represent localized occupational and industrial waste management scenarios rather than environmental background radiation. Therefore, continued regulatory oversight, routine monitoring, and appropriate TENORM management practices are essential to ensure the safe handling, transport, storage, reuse, and disposal of these industrial residues. Published measurements generally indicate low radionuclide concentrations in the investigated groundwater, seawater, and marine biota. However, quantitative radiological risk assessments were not performed for these matrices in the present review. Overall, this study establishes a baseline framework for future research, risk assessment, and policy development concerning environmental radionuclides in arid and semi-arid regions.

## Supplementary Information

Below is the link to the electronic supplementary material.

Supplementary file1 (DOCX 43 kb)

## Data Availability

No datasets were generated or analysed during the current study.
