# Supplementary material for: Risk assessment of radionuclides in different environmental matrices in the State of Qatar, Arabian Gulf
Source: Environ Geochem Health. 2026 Sep 9;48(14):582. doi: 10.1007/s10653-026-03469-1 (PMC13558424; doi:10.1007/s10653-026-03469-1)
Supplement: Supplementary file 1 [file 10653_2026_3469_MOESM1_ESM.docx]

**Supporting information**

**Risk assessment of radionuclides in different environmental matrices in the State of Qatar, Arabian Gulf**

Veerasingam Subramanian^1^*, Vinothini Vinoba^1^, Ranjani Maruthavanan^1^, Rania Edrees Adam Mohammad^1^, Suresh G^2^, Rajendran S^1^, Kishor Kumar Sadasivuni^3^, Saud Ghani^4^, Fatima Al-Khayat^5^

^1^Environmental Science Center, Qatar University, Doha, P.O Box 2713, Qatar

^2^Department of Humanities and Sciences/Physics Division, Aarupadai Veedu Institute of Technology, Vinayaka Mission's Research Foundation (DU), Payanoor – 603 104, Chennai, Tamil Nadu, India

^3^Center for Advanced Materials, Qatar University, PO Box 2713, Doha, Qatar

^4^Mechanical and Industrial Engineering, Qatar University, Doha – 2713, Qatar

^5^Department of Biological and Environmental Sciences, College of Arts and Sciences, Qatar University, Doha, Qatar

Email: [v.subramanian@qu.edu.qa](mailto:v.subramanian@qu.edu.qa)

Table S1. Comparison of radionuclide concentrations in Qatar with global mean concentrations

| **Matrix** | **Radionuclide** | **Range in Qatar** | **Global Mean concentrations level** | **Sources** |
| --- | --- | --- | --- | --- |
| Soil | ^226^Ra | 5–350 Bq/kg | 33 Bq/kg | UNSCEAR, 2000 |
|  | ^238^U | 2.46–213.9 Bq/kg | 33 Bq/kg | UNSCEAR, 2000 |
|  | ^232^Th | 0.42–20 Bq/kg | 45 Bq/kg | UNSCEAR, 2000 |
|  | ^40^K | 10–327 Bq/kg | 420 Bq/kg | UNSCEAR, 2000 |
|  | ^137^Cs | <MDA – 47 Bq/kg | 2 -20 Bq/kg | Global fallout synthesis (2008-2022) |
|  | ^90^Sr | 1 – 5.49 Bq/kg | 1 – 5 Bq/kg | - |
|  | ^238^Pu | <0.0160 – 0.0266 Bq/kg | 0.01 – 0.05 Bq/kg | IAEA/UNSCEAR fallout data |
|  | ^239^Pu | 0.042 – 0.261 Bq/kg | 0.02 -0.05 Bq/kg | UNSCEAR, 2000 |
|  | ^240^Pu | 0.027 – 0.258 Bq/kg | 0.05– 0.25 Bq/kg | UNSCEAR, 2000 |
| Sediment | ^228^Ra | 1–6 Bq/kg | 30 Bq/kg | UNSCEAR, 2000 |
|  | ^226^Ra | 4.2–19.5 Bq/kg | 32 Bq/kg | UNSCEAR, 2000 |
|  | ^238^U | 36.2 ± 1.9 – 49.3 ± 3.0 Bq/kg | 33 Bq/kg | UNSCEAR, 2000 |
|  | ^232^Th | 1 – 6 Bq/kg | 45 Bq/kg | UNSCEAR, 2000 |
|  | ^40^K | 11 – 188 Bq/kg | 420 Bq/kg | UNSCEAR, 2000 |
|  | ^137^Cs | 0.12 – 0.66 Bq/kg | 1-2 Bq/kg | Global fallout synthesis (2008-2022) |
| Seawater | ^137^Cs | 0.89 – 2.31 Bq/m^3^ | 1-3 Bq/m^3^ | UNSCEAR, 2000 |
| Groundwater | ^222^Rn | 2.71 – 60.7 Bq/L | 11.1 Bq/L | USEPA1, 2011 |
| Building Materials | ^228^Ra | 4.5–128 Bq/kg | 50 Bq/kg | UNSCEAR, 2000 |
|  | ^226^Ra | 3.5–399 Bq/kg | 35 Bq/kg | UNSCEAR, 2000 |
|  | ^235^U | 0.4 – 2.8 Bq/kg | 0.7 to 1.2 Bq/kg | UNSCEAR, 2000 |
|  | ^232^Th | 0.38 – 183 Bq/kg | 45 Bq/kg | UNSCEAR, 2000 |
|  | ^234^Th | 4.7 – 41.8 Bq/kg | 33 Bq/kg | UNSCEAR, 2000 |
|  | ^40^K | 0.5 – 242 Bq/kg | 500 Bq/kg | UNSCEAR, 2000 |
|  | ^137^Cs | 0.1 – 1.4 Bq/kg | ~1 Bq/kg | UNSCEAR, 2000 |
| Biota | ^40^K | 21.3–143 Bq/kg | 100-130 Bq/kg | UNSCEAR, 2000 |
|  | ^137^Cs | 0.04 – 0.42 Bq/kg | 0.5 Bq/kg | UNSCEAR, 2000 |

Table S2. Radiological risk indices for soil, sediment and building materials in Qatar

| **Sample Type** | **^226^Ra**  **(Bq/kg)** | **^232^Th**  **(Bq/kg)** | **^40^K**  **(Bq/kg)** | **Ra_eq_**  **(Bq/kg)** | **D**  **(nGy/h)** | **AEDE**  **(mSv/y)** | **AGDE**  **(µSv/y)** | **H_ex_** | **H_in_** | **ELCR**  **(10^-3^)** | **References** |
| --- | --- | --- | --- | --- | --- | --- | --- | --- | --- | --- | --- |
| Soil | 17.20 | 6.38 | 169.00 | 39.34 | 18.90 | 0.023 | 132.88 | 0.106 | 0.153 | 0.081 | Al-Sulaiti et al. 2017 |
| Soil | 32.00 | 18.00 | 327.00 | 82.92 | 39.39 | 0.048 | 276.80 | 0.224 | 0.310 | 0.169 | Ahmad et al. 2019 |
| Soil | 29.50 | 11.00 | 320.00 | 69.87 | 33.71 | 0.041 | 237.62 | 0.189 | 0.268 | 0.145 | Al-Sulaiti et al. 2012 |
| Soil | 20.05 | 16.43 | 216.69 | 60.23 | 28.29 | 0.035 | 198.67 | 0.163 | 0.217 | 0.121 | Al-Kinani et al. 2015a |
| Contaminated soil | 238.17 | 8.43 | 114.40 | 259.03 | 119.93 | 0.147 | 807.10 | 0.700 | 1.344 | 0.515 | Al-Kinani et al. 2015a |
| Sludge | 14577.53 | 65.15 | 97.35 | 14678.19 | 6778.26 | 8.313 | 45347.46 | 39.671 | 79.069 | 29.095 | Al-Kinani et al. 2015a |
| Soil | 32.02 | 8.88 | 207.34 | 60.68 | 28.87 | 0.035 | 201.16 | 0.164 | 0.250 | 0.124 | Al-Kinani et al. 2012b |
| Sediment | 7.40 | 3.50 | 50.60 | 16.30 | 7.66 | 0.009 | 53.38 | 0.044 | 0.064 | 0.033 | Al-Qaradawi et al. 2015 |
| Qatari cement | 23.40 | 12.20 | 158.80 | 53.07 | 24.85 | 0.030 | 173.17 | 0.143 | 0.207 | 0.107 | Al-Sulaiti et al. 2011 |
| Sand | 13.20 | 3.34 | 225.50 | 35.34 | 17.59 | 0.022 | 125.56 | 0.095 | 0.131 | 0.075 | Al-Sulaiti et al. 2011 |
| Washed sand | 13.50 | 3.60 | 228.20 | 36.22 | 18.00 | 0.022 | 128.42 | 0.098 | 0.134 | 0.077 | Al-Sulaiti et al. 2011 |
| White cement | 18.90 | 4.90 | 62.90 | 30.75 | 14.33 | 0.018 | 98.63 | 0.083 | 0.134 | 0.062 | Al-Sulaiti et al. 2011 |
| Clinker | 23.50 | 9.70 | 185.00 | 51.62 | 24.49 | 0.030 | 171.25 | 0.139 | 0.203 | 0.105 | Al-Sulaiti et al. 2011 |
| Klin feed | 15.30 | 6.80 | 139.00 | 35.73 | 17.01 | 0.021 | 119.35 | 0.097 | 0.138 | 0.073 | Al-Sulaiti et al. 2011 |
| Gypsum | 11.50 | 2.50 | 72.00 | 20.62 | 9.85 | 0.012 | 68.59 | 0.056 | 0.087 | 0.042 | Al-Sulaiti et al. 2011 |
| Road building material | 8.10 | 1.53 | 14.30 | 11.39 | 5.27 | 0.006 | 35.91 | 0.031 | 0.053 | 0.023 | Al-Kawari & Hushari 2019 |
| Pure slag from Qatar steel | 283.40 | 166.60 | 9.32 | 522.36 | 231.95 | 0.284 | 1575.02 | 1.411 | 2.177 | 0.996 | Al-Kawari & Hushari 2019 |
| Gabbro | 6.71 | 1.27 | 13.14 | 9.54 | 4.42 | 0.005 | 30.16 | 0.026 | 0.044 | 0.019 | Al-Kawari & Hushari 2019 |
| Slag | 273.18 | 135.18 | 12.51 | 467.44 | 208.38 | 0.256 | 1413.08 | 1.263 | 2.001 | 0.894 | Al-Kawari & Hushari 2019 |
| Steel slag | 324.60 | 111.70 | 10.00 | 485.10 | 217.85 | 0.267 | 1473.06 | 1.311 | 2.188 | 0.935 | Al-Kinani et al. 2015b |
| Asphalt Mix / Concrete Cube | 220.00 | 62.25 | 67.63 | 314.23 | 142.08 | 0.174 | 961.24 | 0.849 | 1.444 | 0.610 | Al-Kinani et al. 2015b |
| Road asphalt and concrete | 25.50 | 9.55 | 42.75 | 42.45 | 19.34 | 0.024 | 132.14 | 0.115 | 0.184 | 0.083 | Al-Kinani et al. 2015b |

Table S3. Recommended values for radiological parameters

| Radiation indices | Recommended values (UNSCEAR, 2000) |
| --- | --- |
| Radium equivalent activity (Ra_eq_) | 370 Bq/kg |
| Absorbed gamma dose rate | 59 nGy/h |
| Annual effective dose equivalent | 0.07 mSv/y |
| Annual gonadal dose equivalent | 300 µSv/y |
| External hazard index (H_ex_) | <1 |
| Internal hazard index (H_in_) | <1 |
| Excess lifetime cancer risk (ELCR) | 0.29 × 10^-3^ |
